# Supplementary material for: Continuous biomarker monitoring by particle mobility sensing with single molecule resolution
Source: Nat Commun. 2018 Jun 29;9:2541. doi: 10.1038/s41467-018-04802-8 (PMC6026194; doi:10.1038/s41467-018-04802-8)
Supplement: Supplementary file 1 — Supplementary Information [file 41467_2018_4802_MOESM1_ESM.pdf]

Supplementary Information

Continuous Biomarker Monitoring by Particle Mobility Sensing with Single Molecule Resolution

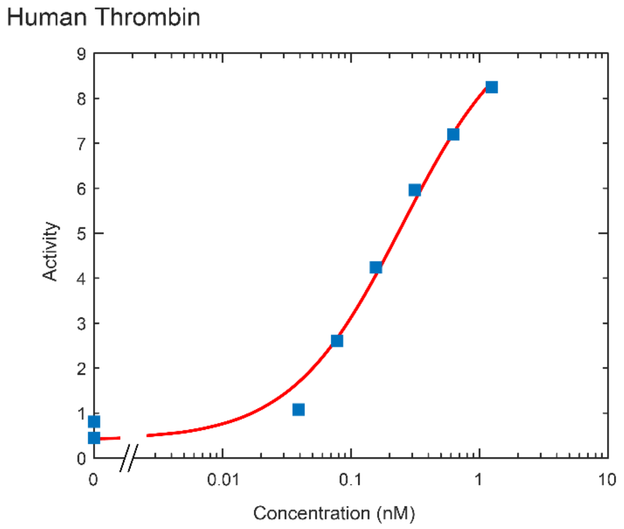

Supplementary Figure 1: Dose-response curve for human thrombin. *Particle switching activity for the experimental system of Fig. 3c, now with thrombin from human plasma (T1063, Sigma Aldrich) as the target in solution. The red line shows the Hill equation fit, which gives* $K_d = 250 \pm 60$ *pM. This is close to the* $K_d = 500\ pM$ *reported in literature for the interaction between human thrombin and the used 29-nt aptamer.*[1] *Due to the low* K_d *for human thrombin, very low concentrations can be detected, but on the other hand the relaxation times are long (longer than the duration of the experiment).*

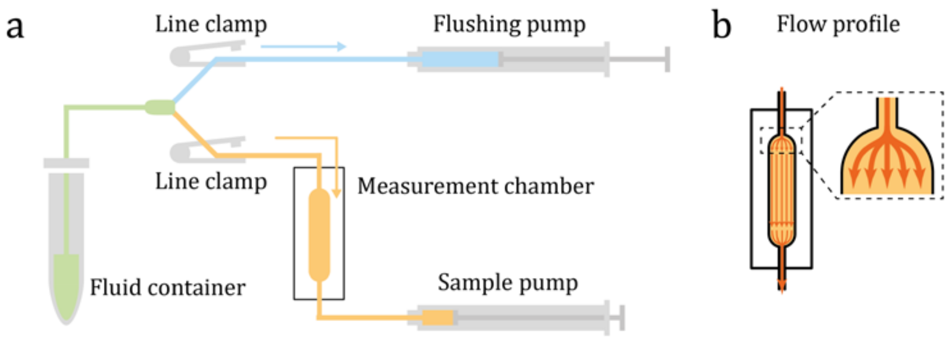

Supplementary Figure 2: Schematic representation of the flow system. *(a) Sample fluid (green) is aspirated from the sample container using a tube. The tube is split using a Y-connector into the blue line, which is used for flushing, and the orange line, which is used to flow the fluid through the measurement chamber. Two syringe pumps are used to control the fluid flow through both systems. The two clamps can be closed individually to prevent unintended flow in the lines. (b) Sketch of the flow profile observed through the measurement chamber.*

Supplementary Note 1: Error in measurement of switching activity

The switching activity reported in Fig. 3 has two main error contributions. The first is the stochastic noise originating from the stochastic rate at which the reactions occur. The stochastic error $\sigma_{stochastic}$ in the activity (expressed as number of mobility change events per particle, per measurement time) in a single experiment is given by:

$$\sigma_{\text{stochastic}} = \frac{\sqrt{N_{\text{events,total}}}}{N_{\text{particle}}} = \frac{\sqrt{\text{Activity}}}{\sqrt{N_{\text{particle}}}}. \tag{1}$$

In our experiments, where several hundred particles are used to determine the activity, the relative stochastic error is on the order of a few percent:

$$\frac{\sigma_{\text{stochastic}}}{\text{Activity}} = \frac{1}{\sqrt{\text{Activity} \cdot N_{\text{particle}}}} = \frac{1}{\sqrt{N_{\text{events,total}}}}. \tag{2}$$

The second error contribution originates from experimental uncertainties. This includes:

1. Uncertainty in the exact time that the target concentration enters the measurement chamber.

2. Variation in the handling time (~1 minute) between fluid inflow and start of the measurement. As the measurements are not made in equilibrium and the activity is changing with time, this leads to some variation in the activity measured as a function of the target concentration.

3. Particles can become (temporarily) non-responsive due to non-specific interactions. This effect is especially significant when an above-average active particle becomes non-responsive.

4. Particles can also become non-responsive when bound with multiple interactions, leading to bond lifetimes longer than the measurement time.

5. Particles can become detached due to the fluid flow sheer stress (a particle loss of about 30% is observed over the course of an experiment of several hours, which we attribute to dissociation of antibody-antigen bonds).

Supplementary Note 2: Lifetime of the bound state

Figs. 2 and 4 report lifetimes measured in BPM experiments with DNA. Here we compare the measured bound state lifetimes to numbers reported in literature. Strunz *et al.* determined force-induced dissociation rates of oligo-oligo interactions using AFM dynamic force spectroscopy.[2] Extrapolation to zero force gives an estimation of the thermal dissociation rate, resulting in a semi-empirical relationship for the dissociation rate constant for an oligo-oligo interaction:

$$k_{off} = 10^{3-0.5n}, \tag{3}$$

with n the number of complementary consecutive nucleotides, at room temperature in PBS buffer for 60% GC-content. [2] This predicts a lifetime of 30 s for a 9 bp bond. Single-molecule fluorescence data show a lifetime of 10 s for a 9 bp bond.[3] The DNA experiment in Fig. 2 (9 bp complementarity) gives a lifetime of 7 s, which is comparable to the single-molecule lifetimes reported in literature.

Supplementary Note 3: Dependence of lifetime of the unbound state on target concentration

Fig. 2c shows the dependence of the unbound state lifetimes on the target concentration. The unbound state lifetimes correspond to an association rate of the target-laden particle to the substrate. The unbound state lifetimes decrease when more target molecules are captured on the particle. The observed unbound state lifetimes scale approximately as $\sim[\mathrm{T}]^{-1/2}$ for the DNA and thrombin experiments (see Fig. 2). In the limit of low target concentration, a simple first-order reaction kinetics model would predict a scaling as $\propto [\mathrm{T}]^{-1}$. Here we discuss possible origins of the observed behavior:

1. Target concentrations above the K_{d} of the interaction cause a saturation of the capture molecules on the particle. This would give a scaling $\propto [\mathrm{T}]^{-n}$ with n below 1, and with n approaching zero at concentrations well above the K_{d}.

2. Particles with an above-average activity have a higher chance of becoming bound to the substrate with multiple target molecules. These particles are then, on the 5 min timescale of the experiment, irreversibly bound. This leads to a relatively lower observed activity of the particles at higher target concentrations, lowering the observed scaling exponent n.

Supplementary Note 4: Relaxation time

Here we estimate the relaxation time of the BPM system based on first-order reaction kinetics. The capture molecules on the particle have the strongest affinity to the target molecules, so the interaction between capture molecules and target molecules determines the relaxation time of the system. The switching activity of the system depends on the occupancy of capture molecules (here referred to as A) by target molecules (referred to as T), i.e. on the formation of A-T complexes on the particle. In case of a constant target concentration in solution ($[\mathrm{T}] = T_0$), the differential equation for the formation of A-T complexes is:

$$\frac{d[\mathrm{AT}]}{dt} = k_{on}[\mathrm{A}]T_0 - k_{off}[\mathrm{AT}] = k_{on}(A_0 - [\mathrm{AT}])T_0 - k_{off}[\mathrm{AT}] \tag{4}$$

where k_{on} is the association rate constant, k_{off} is the dissociation rate constant, and A_0 is the starting concentration of capture molecules. This rearranges to the linear differential equation:

$$\frac{d[\mathrm{AT}]}{dt} + \left(k_{off} + k_{on}T_0\right)[\mathrm{AT}] = k_{on}A_0T_0 \tag{5}$$

In equilibrium ($d[\mathrm{AT}]/dt = 0$), the equation simplifies to:

$$f_{AT} = \frac{[\mathrm{AT}]}{A_0} = \frac{T_0}{K_d + T_0} \tag{6}$$

where f_{AT} is the fractional occupancy of capture molecules by target molecules and $K_d = k_{off}/k_{on}$ is the equilibrium dissociation constant. When at $t = 0$ a concentration step is applied from $[\mathrm{T}] = T_0$ to $[\mathrm{T}] = T_1$, the solution of the differential equation is:

$$f_{AT}(t) = \frac{T_0}{K_d + T_0} + \left(\frac{T_1}{K_d + T_1} - \frac{T_0}{K_d + T_0}\right)\left(1 - e^{-t/\tau}\right) \tag{7}$$

where $f_{AT}(t)$ is the fractional occupancy of capture molecules by target molecules as a function of time and $\tau = 1/\left(k_{off} + k_{on}T_1\right)$ is the time constant that describes the characteristic time with which the system reaches equilibrium.

We can now compare the relaxation data of Fig. 3 to values from literature. We focus on the DNA experiment because literature values are available for oligo-oligo interactions. The DNA experiment in Fig. 3b shows decay characteristics with $\tau_{off} = 35$ min. In that experiment, a target-free buffer was supplied ($T_1 = 0$), so $\tau = 1/k_{off}$ (see Supplementary Equation 7). The capture oligo in the experiment of Fig. 3 has 11 nucleotides complementary to the target molecule, so the semi-empirical equation by Strunz *et al.*[2] yields an estimated $k_{off} = 3.2 \cdot 10^{-3}$ s^{-1}, which corresponds to a characteristic dissociation time $\tau_{off} = 1/k_{off}$ of 5 min. Therefore, the relaxation time observed for the DNA system in the BPM experiment is much longer than the characteristic thermal dissociation time estimated from the AFM experiments by Strunz *et al.* One possible explanation is that the close proximity of particle and substrate in the BPM experiment causes a nonzero target rebinding probability that slows down the diffusive target escape process.[4]

Supplementary Note 5: Cartridge and flow system

A flow system was used to sequentially introduce the antibodies, oligonucleotides, and particles into the measurement chamber, and to perform the washing steps during the preparation of the sample. During measurements, the solution with target molecules was introduced into and flushed out of the system in a volume and flow-speed controlled manner. We used an in-house built setup (components from TLG, Billund, Denmark) for organizing the tubing, solutions, connectors and the sample. Two electronic, programmable syringe pumps (Oxford Instruments, UK) were used to control the fluid flow. Tubes were closed with clamps when no flow was required.

The measurement chambers were constructed on a glass microscope slide (75x25 mm #5, Menzel-Gläser, Germany). The glass was cleaned using two 10-minute sonication treatments in a sonic bath. The first sonication step was performed in isopropyl alcohol and the second in methanol. After that, the microscope slides were dried using a gentle stream of nitrogen. A flow cell sticker (Grace Biolabs, USA) was attached to the glass substrate to form the flow channel. The flow channel has a custom design of a 20 mm long, 4 mm wide chamber with a depth of approximately 300 μm. The top side of the flow cell sticker was made from optically clear plastic, 200 μm thick. At the two ends of the flow channel, two flexible tubes were inserted into the flow channel through 1 mm holes in the top plastic. The tubes were fixed in place using Ultra Light-Weld 142-M glue (Dymax, Wiesbaden, Germany). The glue was cured using a 10 second UV exposure with an OmniCure S1000 (Lumen Dynamics, Canada) UV-exposure setup. The placement of the tubes at the end of the flow channel ensures quick development of a homogeneous, laminar flow profile over the entire flow channel with a minimal dead volume.

A schematic representation of the flow system is shown in Supplementary Figure 2. The flow system was used to control the routing, aspiration, and flushing of fluids through the measurement chamber. A piece of silicon tubing (inner diameter 0.76 mm, Helixmark tubing, Freudenberg medical, Germany) was connected to a needle, which was placed into the solution that was to be aspirated into the flow system. The aspiration tubing was connected to a vertically placed three-way Y-shaped connector, which connects the aspiration tubing to two separate lines. The Y-connector had a small internal volume (20 μL), which served as a trap for small air bubbles that may be introduced into the system due to switching between different fluids. A piece of silicon tubing was connected to the upper arm of the Y-connector and then fed through a clamp. The clamp could be engaged and disengaged as required to compress the silicon tube and block fluid flow. The silicon tube was then connected to the syringe pump which actuated the fluid flow. This line is referred to as the blue line. In addition to flushing the tubing with fluid, the blue line could be used to remove air bubbles that became trapped in the Y-connector. A third piece of silicon tubing formed the second line (the orange line), which was fed through the second clamp, and then connected to the measurement chamber. A final piece of silicon tubing connected the measurement chamber with the second syringe pump to complete the orange line.

To ensure that all fluid in the measurement chamber was exchanged effectively, both fluid connections were made at the apices of the rounded ends of the flow cell. The resulting flow profile is shown in Supplementary Figure 2b. The flow profile was observed using the suspended particles during typical flow steps. No dead volumes or vortices were observed in the measurement chamber at either of the two ends.

Supplementary References

1. Tasset, D. M., Kubik, M. F. & Steiner, W. Oligonucleotide inhibitors of human thrombin that bind distinct epitopes. *J. Mol. Biol.* **272,** 688–698 (1997).

2. Strunz, T., Oroszlan, K., Schäfer, R. & Güntherodt, H. J. Dynamic force spectroscopy of single DNA molecules. *Proc. Natl. Acad. Sci. U. S. A.* **96,** 11277–11282 (1999).

3. Johnson-Buck, A. *et al.* Kinetic fingerprinting to identify and count single nucleic acids. *Nat. Biotechnol.* **33,** 730–732 (2015).

4. Lagerholm, B. C. & Thompson, N. L. Theory for Ligand Rebinding at Cell Membrane Surfaces. *Biophys. J.* **74,** 1215–1228 (1998).
