# Supplementary material for: Continuous biomarker monitoring by particle mobility sensing with single molecule resolution
Source: Nat Commun. 2018 Jun 29;9:2541. doi: 10.1038/s41467-018-04802-8 (PMC6026194; doi:10.1038/s41467-018-04802-8)
Supplement: Supplementary file 3 — Description of Additional Supplementary Files [file 41467_2018_4802_MOESM3_ESM.pdf]

Description of Additional Supplementary Files

File Name: Supplementary Movie 1
Description: Animation of Biosensing by Particle Mobility. Sketched are: particles (orange). dsDNA tether (blue helix), capture molecules on the particle (blue), capture molecules on the substrate (purple), biomarker molecules in solution (green).
